# A retrospective study of adverse effects of mycophenolate mofetil administration to dogs with immune‐mediated disease

**DOI:** 10.1111/jvim.16209

**Published:** 2021-07-06

**Authors:** Kenjiro Fukushima, Michael Lappin, Marie Legare, Julia Veir

**Affiliations:** ^1^ Department of Clinical Sciences Colorado State University Fort Collins Colorado USA; ^2^ Department of Environmental and Radiological Health Science Colorado State University Fort Collins Colorado USA

**Keywords:** adverse events, canine, immunosuppressant, toxicity

## Abstract

**Background:**

Information regarding adverse events (AEs) of mycophenolate mofetil (MMF) is limited.

**Objectives:**

To evaluate the types and frequency of potential AEs of MMF in dogs with immune‐mediated disease.

**Animals:**

One hundred thirty‐one dogs treated with MMF for management of suspected immune‐mediated disease.

**Methods:**

Retrospective study. Medical records were reviewed to find and group suspect AEs in gastrointestinal (GI), hematologic, and other categories. Age, dosage, body weight, and sex were analyzed between dogs with and without AEs by using the Mann‐Whitney *U*‐test and chi‐squared test.

**Results:**

The median starting dosage of MMF was 17.5 mg/kg/day (interquartile range [IQR] = 15.1‐20.6 mg/kg/day) and the median treatment duration was 56 days (IQR = 14‐236 days). Mycophenolate mofetil was prescribed for immune‐mediated hemolytic anemia (n = 31), immune‐mediated thrombocytopenia (n = 31), pemphigus foliaceus (n = 15), immune‐mediated polyarthritis (n = 12), and others (n = 42). Overall, potential AEs of MMF were observed in 34 of 131 dogs (GI 24.4% [31/127], neutropenia 4% [3/76], anemia 4% [1/25], thrombocytopenia 4.0% [1/25], and dermatologic 1.5% [2/131]). There were no significant differences among dogs with (n = 37) or without potential AEs (n = 94) in regards to sex, age, body weight, or dosage of MMF (*P* = .06, .13, .24, and .26, respectively).

**Conclusions and Clinical Importance:**

In the dogs administered MMF, GI AEs were most common. Since potential hematologic and dermatologic AEs developed in a few dogs, clinicians should be aware of these when prescribing MMF to dogs with immune‐mediated disease.

**ABBREVIATIONS**
VTH: Veterinary Teaching Hospital
GI: gastrointestinal
IMHA: immune‐mediated hemolytic anemia
IMNP: immune‐mediated neutropenia
IMPA: immune‐mediated polyarthritis
ITP: immune‐mediated thrombocytopenia
MMF: mycophenolate mofetil

## 1 INTRODUCTION

Mycophenolate mofetil (MMF) is an immunosuppressant used for the management of immune‐mediated diseases in dogs.^1^, ^2^, ^3^, ^4^, ^5^, ^6^, ^7^ A metabolite of MMF, mycophenolic acid (MPA), selectively and reversibly inhibits inosine 5′‐monophosphate dehydrogenase (IMPDH), which is a rate limiting enzyme of de novo synthesis of the nucleotide guanine.^1^ Since rapidly growing cells like lymphocytes have a high demand for guanine nucleotide, inhibition of IMPDH results in arrest of proliferation of both T and B lymphocytes and consequently causes immunosuppression. In dogs, MMF is prescribed for treatment of immune‐mediated thrombocytopenia (IMTP) and immune mediated hemolytic anemia (IMHA) and, based on consensus guidelines, is recommended in dogs with glomerulonephritis.^2^, ^3^, ^4^, ^5^, ^8^

There are few publications that describe adverse events from MMF administration to dogs. Diarrhea, vomiting, and inappetence are the most frequently reported. In an experimental study in dogs, dose‐limiting gastrointestinal (GI) adverse events (AEs) from MMF occurred at a dose of 60 mg/kg/day.^9^ Additionally, MMF‐induced GI AEs were noted in a case series of 5 dogs with IMHA in which the median dose of MMF administered was 36.9 mg/kg/day (range, 27.8‐41.1 mg/kg/day).^3^ Diarrhea occurred in 17% of 30 dogs with IMHA that were administered MMF at a median dose of 20.5 mg/kg/day.^2^ In a case series of 5 dogs with ITP, when MMF was prescribed as a single agent at a median dose of 17 mg/kg/day, 2 of the dogs had diarrhea and inappetence.^4^ In a study of 25 dogs with meningoencephalomyelitis that were administered MMF at a mean dose of 20.05 mg/kg/day, 2 dogs developed vomiting and decreased appetite.^7^ In a prospective study in 10 dogs with sudden acquired retinal degeneration syndrome that were administered MMF at a dosage of 20 mg/kg/day, 2 dogs experienced GI adverse events that resolved with dosage reduction of MMF by 20%.^10^ It was reported that 6 (42.9%) of 14 dogs with immune‐mediated skin disease that were treated with MMF at mean dosage of 29.4 mg/kg/day experienced GI AEs.^6^ Although these studies describe AEs from MMF at a wide range of doses, the number of the cases in most reports was low and case details were not well documented. In humans, females have significantly higher GI adverse event scores compared to males suggesting a sex‐related effect.^11^ In addition, adverse events in dogs affecting body systems other than the GI system have not been well described. In the study of immune‐mediated skin disease treated with MMF in 14 dogs, hepatotoxicity or bone marrow suppression was not observed.^6^ Other veterinary studies have not specifically examined MMF associated adverse events in other body systems in detail.

The purpose of the current study is to describe the types and frequency of adverse events likely to be associated with administration of MMF to dogs with naturally occurring immune‐mediated diseases.

## 2 MATERIALS AND METHODS

### 2.1 Case selection and medical records review

Medical records of dogs that were prescribed MMF at a Veterinary Teaching Hospital (VTH) between January 2011 and July 2018 were identified by computerized medical record review. Dogs were excluded from the study if no follow‐up was available or if the dog was euthanized immediately following diagnosis. Data extracted from the medical record included signalment, body weight, primary disease diagnoses, starting dosage of MMF, treatment duration, concurrent medications, and potential adverse events. Dogs lacking a definitive diagnosis were excluded from the study.

Diagnosis of primary IMHA was made based on ACVIM consensus guidelines (signs of immune‐mediated destruction such as positive saline agglutination and spherocytosis, signs of hemolysis such as hyperbilirubinemia, icterus, hemoglobinemia, hemoglobinuria, or erythrocyte ghosts).^12^ Dogs were diagnosed with IMHA if they had ≥2 signs of immune‐mediated destruction or they had ≥1 sign of immune‐mediated destruction and ≥ 1 sign of hemolysis. Underlying diseases were excluded based on a review of the history, imaging (thoracic radiographs and abdominal ultrasound), and at minimum, each dog was negative for antibodies against *Anaplasma* spp., *Ehrlichia* spp., *Dirofilaria immitis*, and *Borrelia burgdorferi* (SNAP 4Dx Plus IDEXX, Westbrook, Maine). Confirmation of the diagnosis of primary ITP was made using the criteria of a platelet count <40 000/μL absence of platelet clumps, and no evidence of underlying disease based on a review of the history, thoracic radiographs, abdominal ultrasound, and each dog was negative for antibodies against *Anaplasma* spp., *Ehrlichia* spp., *Dirofilaria immitis*, and *Borrelia burgdorferi* (SNAP 4Dx Plus IDEXX, Westbrook, Maine).^13^ Pemphigus foliaceous was diagnosed based on the presence of typical clinical signs, lesion distribution, and typical histopathological, cytological findings, or both.^14^ Immune‐mediated polyarthritis (IMPA) was diagnosed based on clinical signs and consistent results of cytology of synovial fluid analysis.^15^ Underlying diseases were excluded based on a review of the history, thoracic radiographs, abdominal ultrasound, and each dog was negative for antibodies against *Anaplasma* spp., *Ehrlichia* spp., *Dirofilaria immitis*, and *Borrelia burgdorferi* (SNAP 4Dx Plus IDEXX, Westbrook, Maine). Diagnostic tests used to obtain other diagnoses are listed in Table A1.

### 2.2 Potential mycophenolate‐related adverse events

Gastrointestinal adverse events (hyporexia, anorexia, vomiting, or diarrhea) possibly attributable to MMF were identified based on medical record description, communication logs in the VTH electronic medical record, or both. These signs were recorded if recognized at any time after MMF administration. Data from dogs with previously diagnosed chronic enteropathies were excluded from the analysis of GI adverse events. Potential hematologic adverse events (anemia, neutropenia, or thrombocytopenia) were evaluated in any dog for which CBC data were available before (within 1 week of the initiation of therapy) and after starting therapy with MMF (at least 5 days). Anemia was defined as PCV < 36% and data from dogs with IMHA, ITP, or systemic lupus erythematosus (SLE) were excluded from this analysis. Neutropenia was defined as neutrophil count <3000/μL and from dogs diagnosed with immune‐mediated neutropenia (IMNP) or SLE were excluded from this analysis. Thrombocytopenia was defined as platelet count <200 000/μL and data from dogs diagnosed with IMHA, ITP, or SLE were excluded from this analysis.

### 2.3 Statistical analyses

All analyses were performed using commercial software (Prism9, GraphPad, San Diego, California). Statistical significance was set as *P* ≤ .05. Data were tested for normal distribution by the D'Agostino and Pearson omnibus normality test. Age, dosage, body weight, and sex were analyzed between dogs with and without adverse events. The Mann‐Whitney *U*‐test and chi‐squared test were used for continuous variables (age, dosage, and body weight) and categorical variables (sex), respectively.

## 3 RESULTS

### 3.1 Animals

Medical records of 181 dogs that were prescribed MMF at the VTH from 2012 to 2018 were reviewed for inclusion in the study. Of these, 50 dogs were excluded because of no follow‐up (n = 28), euthanasia, or death within 3 days after the diagnosis (n = 18), or lack of definitive diagnosis of primary disease (n = 4). One‐hundred thirty‐one dogs were included in this retrospective study. Demographic data is summarized in Table 1.

**TABLE 1:** Demographic data for 131 dogs with mycophenolate

Mycophenolate dosage	17.5 mg/kg/day (15.1‐20.6)
Mycophenolate duration	56 days (14‐236)
Age	7 years (5–10)
Body weight	27.0 (21.7‐35.0)
Sex	
Male neutered	50
Male intact	6
Female spayed	70
Female intact	5
Breed (n)	
Mixed breed	42
Labrador retriever	18
English bulldog	5
Border collie	5
Boxer	5
Standard poodle	5
Australian heeler	4
Golden Retriever	4
Bernese mountain dog	3
Newfoundland	3
German Shepherd dog	3
Australian sheepdog	3
Other breeds	31
Primary disease (n)	
IMHA	31
ITP	31
Pemphigus foliaceus	15
IMPA	13
Immune‐mediated neuromuscular disease	8
Non‐regenerative IMHA	4
ITP/IMHA	4
PLN	4
IBD	4
SLE	3
IMNP	3
Uveitis	3
DLE	3
Others	5

*Note*: Data are reported as medians with interquartile ranges.

### 3.2 Mycophenolate use

The median starting dosage of MMF was 17.5 mg/kg/day (IQR 14.9‐20.4 mg/kg/day). The total daily MMF dose was administered as a single dose in 11 dogs and divided into 2 doses in 120 dogs. The median duration of treatment with MMF was 58 days (IQR 15‐258 days; Table 1). MMF was used as first‐line adjunctive therapy in 67 (51.1%) dogs, second‐line adjunctive therapy in 53 (40.5%) dogs, and first‐line single therapy in 11 (8.4%) dogs. The immunosuppressants being concurrently administered when MMF was added to the treatment protocol were prednisolone alone (n = 107), prednisolone and cyclosporine (n = 5), prednisolone and leflunomide (n = 3), dexamethasone (n = 2), dexamethasone and cyclosporine (n = 1), cyclosporine (n = 1), or budesonide (n = 1). Mycophenolate mofetil was discontinued in 53 (40%) dogs because of adverse events potentially attributed to MMF (n = 20), euthanasia/death because of the severity of the primary disease (n = 16), no response to the primary disease/relapse of the primary disease (n = 11), or remission of the primary disease (n = 6).

## 4 POTENTIAL MYCOPHENOLATE‐RELATED ADVERSE EVENTS

### 4.1 Gastrointestinal

One hundred twenty‐seven dogs met the criteria to be evaluated for potential GI adverse events (IMHA, n = 31; ITP, n = 31; pemphigus foliaceus, n = 15; IMPA, n = 13; immune‐mediated muscular disease, n = 8; non‐regenerative IMHA, ITP/IMHA, and PLN, n = 4 dogs each; SLE, IMNP, uveitis, and discoid lupus erythematosus [DLE], n = 3 dogs each; allergic skin disease and hepatitis, n = 2 each; pancytopenia, n = 1). Gastrointestinal adverse events possibly attributable to MMF were observed in 31 of the included dogs (24.4%, 95% confidence interval [CI] 17.8‐32.6). Among these potential adverse events, diarrhea was the most common (n = 23, 18.1%, 95% CI 12.4‐25.7), followed by hyporexia or anorexia (n = 18, 14.2%, 95% CI 9.2‐21.3), and vomiting (n = 13, 10.1%, 95% CI 6.0‐16.5). The median time to onset of potential GI adverse events was 10 days (IQR; 6‐14 days). Description of fecal consistency was available for 24 of the 31 dogs. Diarrhea was noted in 12 dogs, followed by hematochezia (n = 5), melena (n = 4), and soft stool (n = 3). Veterinary visits prompted by suspected GI adverse events from MMF were noted in 6 cases. In the dogs with reported potential GI adverse events, the drug was discontinued in 15 cases, the dose was reduced in 2 cases, MMF was continued at the same dose in 14 cases, and for 2 cases MMF dose alternations were unclear. Attempt to alter the GI microflora was started in 14 cases (metronidazole: n = 6, tylosin: n = 6, pre/probiotic: n = 2). Follow‐up information after GI adverse events were suspected was available for 28 dogs. Clinical signs were improved in 25 dogs (discontinued MMF in 8 dogs, discontinued MMF with antibiotics in 4 dogs, discontinued MMF with pre/probiotics in 1 dog, reduced dose of MMF in 1 dog, reduced dose of MMF with antibiotics in 1 dog, continued MMF in 5 dogs, continued MMF with antibiotics in 4 dogs, continued MMF with pre/probiotics in 1 dog), not improved in 2 dogs (continued MMF with antibiotics in 2 dogs), and 1 dog was euthanized because of lack of response to primary disease. The median time to improvement was 7 days (IQR: 4‐12 days).

### 4.2 Hematologic

Seventy‐six dogs (IMHA, n = 23; ITP, n = 25; IMPA, n = 6; pemphigus foliaceus, n = 5; IMHA/ITP, IBD, and immune‐mediated neuromuscular disease, n = 3 dogs each; NR‐IMHA, PLN, and uveitis, n = 2; and hepatitis, and DLE, n = 1 dog each) met inclusion criteria to evaluate for neutropenia possibly attributable to MMF administration. The median duration between baseline and the recheck CBC of 15 days (IQR: 9‐35 days). Three dogs developed neutropenia after MMF was prescribed (3.9%, 95% CI 1.1‐11.0). The neutropenia was recognized on days 6, 6, and 55, respectively.

Twenty‐five dogs (pemphigus foliaceus and IMPA, n = 5 dogs each; IMNP, immune‐mediated neuromuscular disease, and IBD, n = 3 dogs each; PLN and uveitis, n = 2 dogs each; and hepatitis and DLE, n = 1 dog each) met inclusion criteria to evaluate for anemia or thrombocytopenia after MMF was prescribed; the recheck CBC was performed a median of 27 days later (IQR: 23‐74 days). One dog developed anemia on day 36 (4.0%; 95% CI 0.2‐19.5). One dog developed mild thrombocytopenia (187 000/μL; no platelet clumping seen) on day 69 (4.0%, 95% CI 0.2‐19.5%).

### 4.3 Dermatologic adverse events

Two dogs developed skin eruptions after start of MMF administration (1.5%, 95% CI 0.3‐5.4).

### 4.4 Statistical analyses

Age, dosage, body weight, and sex were analyzed between dogs with potential adverse events and dogs without adverse events. However, there was no significant difference in age, sex, body weight, and dose between dogs with any potential adverse events associated with MMF (n = 34) and dogs without adverse events (n = 97; Table 2).

**TABLE 2:** Differences between dogs with adverse events and dogs without adverse events possibly associated with the use of mycophenolate mofetil

		Adverse events (n = 34)	No adverse events (n = 97)	*P* ^a^
Age (years)		6 (4‐9)	8 (5–10)	.13
Body weight (kg)		29.1 (21.3‐35.5)	27.0 (21.2‐34.0)	.24
Sex	Male	20	37	.06
	Female	14	60	
MMF dosage (mg/kg/day)		17.6 (15.8‐21.5)	17.4 (14.1‐20.3)	.26

*Note*: Data are reported as median with interquartile range.

^a^ The *P* values in the last column refer to comparison of variables between dogs with adverse events and without adverse events (*P* < .05 is considered to be significant).

## 5 DISCUSSION

In the study described here, MMF administered at the median dose of 17.5 mg/kg/day was potentially associated with GI adverse events in 31 of 127 dogs (24.4%). It remains unclear whether or not all of these potential adverse events were attributable to MMF. However, the potential adverse events occurred relatively soon after MMF treatment was initiated with a median time to onset of 10 days. The potential GI adverse events resolved in 89.3% of cases, sometimes after a discontinuation or dose reduction of MMF or supportive care.

There are a few postulated mechanisms of action for MMF‐induced GI AEs.^1^ One proposed mechanism is that MPA's acyl glucuronide metabolite (acyl‐MPAG) has a cytotoxic effect because of its capacity to form protein adducts. In a previous study, the capacity of human GI cells to produce acyl‐MPAG has been demonstrated.^16^ Secondly, when MMF is hydrolyzed in vivo esterase, MPA and N‐(2‐hydroxyethyl) morpholine are released.^17^ N‐(2‐hydroxyethyl) morpholine has local irritative effects in a rabbit model.^17^ The third possibility is that alterations in the composition of GI microbiota is associated with MMF induced GI AEs in mice.^18^ In the current study, 5 dogs were administered antibiotics or probiotics after they developed potential GI adverse events and all of them responded well without discontinuing or reducing the dose of MMF. Favorable response to antibiotic therapy was also reported in veterinary studies.^3^ These facts support the hypothesis that a dysbiosis secondary to MPA occurs. However, given the fact that potential GI adverse events also resolved in 5 dogs without either a dose reduction of MMF or supportive antibiotic administration, it remains unclear how much GI dysbiosis is contributing the development of potential GI adverse events induced by MMF treatment. Also, there is a possibility that GI AEs (diarrhea, vomiting, and hyporexia) in these dogs were unrelated to MMF administration. There is one case series that describes histopathological intestinal changes secondary to MMF administration in dogs.^3^ In that manuscript, erosive to ulcerative enterocolitis with cryptitis, crypt necrosis were characteristic findings. These findings were nonspecific and can be caused by any of above mechanism of action.

In our study, 3 out of 76 dogs developed neutropenia (3.9%) after initiation of MMF therapy. One of these dogs was receiving azathioprine at the time of initiation of MMF therapy and azathioprine was discontinued at that time. The neutropenia resolved without changing the dose of MMF. Therefore, bone marrow suppression because of MMF was unlikely in this case. However, neutropenia in the remaining 2 cases could be attributable to MMF as one resolved after discontinuation of MMF and the other did not resolve but no other causes were found despite a thorough diagnostic investigation. In humans, MMF‐induced neutropenia occurs in liver transplant patients and hepatitis patients.^19^, ^20^

Anemia after MMF administration was observed in 1 (4.0%) of 25 dogs in our study. Since the dog was also administered prednisolone during MMF therapy, anemia caused by GI bleeding secondary to prednisolone cannot be excluded. Mycophenolate mofetil‐induced red cell aplasia has been reported in human renal transplant patients.^21^ Also, it is reported that MMF inhibits IMPDH in erythroid cells and leading to decreased erythropoiesis in vitro.^22^ However, it is unknown if the anemia observed in the current study is attributable to MMF administration.

Mild thrombocytopenia developed in 1 (4.0%) of 25 dogs after administration of MMF. However, it is less likely that this was MMF induced since it resolved spontaneously without any change in MMF dose. Possible causes of this mild thrombocytopenia include a normal fluctuation or a laboratory error. Mycophenolate mofetil‐associated thrombocytopenia is rarely reported in the human literature.^19^

Potential hepatotoxicosis from MMF treatment could not be evaluated in the current study as 119 (90.8%) out of 131 dogs were given corticosteroids concurrently with MMF. In remaining 12 dogs, liver enzymes were unavailable before and after MMF administration. Hepatotoxicosis is not a commonly reported adverse effect of MMF therapy in humans. In a study of 123 human liver transplant patients in whom MMF was used as a monotherapy to prevent host graft rejection, there was no significant difference in liver enzyme activities before and after 1‐year MMF treatment.^23^

Potential renal toxicosis also could not be assessed in the current study because of lack of available data to evaluate renal function (ie, creatinine, USG, GFR, SDMA, etc.) before and after MMF treatment. In human literature, MMF is preferred over calcineurin inhibitors (ie, cyclosporine), which can cause renal toxicosis in patients with organ transplantation.^23^ Based on this literature, renal toxicosis secondary to MMF administration seems uncommon.

Skin eruption as a potential adverse event from MMF was found in 2 cases in the current study. To the author's knowledge, this is the first veterinary report of potential MMF induced cutaneous drug reaction. Skin biopsy or necropsy was not performed in either dog. Though no further diagnostics were performed and empirical therapy for bacterial pyoderma was started as well, the pattern of lesions and resolution of signs after discontinuation of the MMF in the dog with erosive skin lesions is strongly suggestive of a cutaneous drug reaction.^24^ In humans, dermatologic MMF‐associated adverse effects are also not well documented. There is a single case report that described a suspected case of MMF‐induced severe skin reaction in a person that developing 1 month after initiation of MMF therapy.^25^ Skin biopsy revealed sparse perivascular infiltration of lymphocytes and eosinophils and lesions improved after discontinuation of MMF and initiation of glucocorticoid therapy. Dermatologic issues such as hair loss or rash were noted in 1.8% of human patients in a meta‐analysis.^20^ Even if the reactions described in our study are truly associated with MMF, dermatologic adverse events would still be extremely rare in dogs.

There was no significant difference in age, sex, body weight, and dosage of MMF between dogs with potential adverse events of MMF and dogs without reported adverse events. In humans, it is reported that females have significantly higher GI adverse event scores compared to males. It is suggested that this may relate to sex differences in hepatic glucuronidation or altered enterohepatic circulation. In the study described here, suspected MMF adverse events in male dogs (36.8%, including 3 intact males) were higher than in females (21.6%, including 1 intact female), but the difference was not significantly different. Because there were so few intact animals in the study, differences may not be able to be detected using our data.

There was no difference in dose of MMF between dogs with suspected adverse events and dogs without adverse events. Mycophenolate mofetil has a large interpatient pharmacokinetic variability.^1^ According to previous pharmacokinetic studies in healthy dogs, maximum concentration (*C*_max_) of MMF differs by approximately 6‐fold among dogs and the area under the curve for 12 hours after administration (AUC_0‐12 hours_) differs by 10‐fold among dogs.^9^, ^26^ These large pharmacokinetic variabilities of MMF might explain potential adverse events in some dogs in our study. Based on the variability among dogs, it will likely prove difficult to predict adverse events from MMF in individual dogs. Therefore, it is important to be aware of common and rare adverse events from MMF, when these can happen, what can be used to manage these potential adverse events.

This study has several limitations with the most important being the retrospective nature. The treatment regimen was not uniform for every dog, which made it difficult to evaluate potential adverse events of MMF treatment. In addition, duration of time between rechecks and monitoring variables were variable and potentially led to under or over estimation of potential adverse events from MMF administration. Since data collection was based on descriptions in the medical records and communication log, there is a possibility that clinical adverse events potentially associated with MMF treatment were underestimated. As there are no data regarding blood concentrations of MPA, the relationship between MPA blood concentrations and potential adverse events is unknown.

## CONFLICT OF INTEREST DECLARATION

Authors declare no conflict of interest.

## OFF‐LABEL ANTIMICROBIAL DECLARATION

Off‐label use of metronidazole and tylosin.

## INSTITUTIONAL ANIMAL CARE AND USE COMMITTEE (IACUC) OR OTHER APPROVAL DECLARATION

Authors declare no IACUC or other approval was needed.

## HUMAN ETHICS APPROVAL DECLARATION

Authors declare human ethics approval was not needed for this study.

## Supporting information

**Appendix Table A1:** Diagnoses and diagnostic tests used to obtain diagnoses in dogs treated with MMF (n <10).
