# Erratum for “A retrospective study of adverse effects of mycophenolate mofetil administration to dogs with immune‐mediated disease”

**DOI:** 10.1111/jvim.16493

**Published:** 2022-07-20

**First published: 06 July 2021** https://onlinelibrary.wiley.com/doi/10.1111/jvim.16209

**Volume 35, Issue 5**

 

The SNAP 4Dx Plus IDEXX is an antigen test for *Dirofilaria immitus*, not an antibody test.

 

Section 2.1 Case selection and medical records review, paragraph 2, sentences 3 and 4 are corrected as follows.

 

Underlying diseases were excluded based on a review of the history, imaging (thoracic radiographs and abdominal ultrasound), and at minimum, each dog was negative for antibodies against *Anaplasma spp*., *Ehrlichia spp*., *Borrelia burgdorferi*, **and antigens against *Dirofilaria immitis*** (SNAP 4Dx Plus IDEXX, Westbrook, Maine). Confirmation of the diagnosis of primary ITP was made using the criteria of a platelet count <40 000/μL absence of platelet clumps, and no evidence of underlying disease based on a review of the history, thoracic radiographs, abdominal ultrasound, and each dog was negative for antibodies against *Anaplasma spp*., *Ehrlichia spp*., *Borrelia burgdorferi*, **and antigens against *Dirofilaria immitis*** (SNAP 4Dx Plus IDEXX, Westbrook, Maine).^13^

 

Section 2.1 Case selection and medical records review, paragraph 2, second to last sentence is corrected as follows.

 

Underlying diseases were excluded based on a review of the history, thoracic radiographs, abdominal ultrasound, and each dog was negative for antibodies against *Anaplasma spp*., *Ehrlichia spp*., *Borrelia burgdorferi*, **and antigens against *Dirofilaria immitis*** (SNAP 4Dx Plus IDEXX, Westbrook, Maine).

