# Supplementary material for: A retrospective study of adverse effects of mycophenolate mofetil administration to dogs with immune‐mediated disease
Source: J Vet Intern Med. 2021 Jul 6;35(5):2215–21. doi: 10.1111/jvim.16209 (PMC8478029; doi:10.1111/jvim.16209)
Supplement: Supplementary file 1 — Appendix Table A1: Diagnoses and diagnostic tests used to obtain diagnoses in dogs treated with MMF (n <10). [file JVIM-35-2215-s001.pdf]

Appendix

Table 1. Diagnoses and diagnostic tests used to obtain diagnoses in dogs treated with MMF (n <10).

Disease		Diagnosis proven*
Immune-mediated (IM) neuromuscular diseases (8)	IM-neurological (6)	Routine lab work + infectious testing (Neospora, Toxoplasma) + MRI + CSF (6)
	IM-myositis (2)	EMG + Muscle biopsy (1)<br>Muscle biopsy (1)
Non-regenerative IMHA (4)		Routine lab work (CBC, Chem) + infectious testing (tick borne disease panel) + TXR + AUS (4) & BMA (3), BMB (1)
ITP/IMHA (4)		Diagnostic criteria for IMHA/ITP and lack of evidence of DIC on coagulation testing (PT, aPTT, D-dimer, FDP)
PLN (4)		Routine lab work (Chem, UA) + infectious testing (tick borne disease panel) + blood pressure + UPC (4)
IBD (4)		Routine lab work (CBC, Chem, UA) + fecal exam/deworming + diet trial + GI biopsy (4)
SLE (3)		Routine lab work (CBC, Chem, UA) + infectious testing (tick borne disease panel) + TXR + AUS + arthrocentesis (3)<br>Cytopenia + IMPA + proteinuria (1)<br>Cytopenia + IMPA + skin biopsy (1)<br>Cytopenia + IMPA + skin lesion (1)
IMNP (3)		Routine lab work (CBC, Chem) + infectious disease testing (tick borne disease panel) + TXR + AUS + BMA (3)
Uveitis (3)		Routine lab work (CBC, Chem, UA) + infectious disease testing (tick borne disease panel) + TXR + AUS + ophthalmic exam (3)
DLE (3)		Routine lab work (CBC, Chem, UA) + Skin biopsy (3)
Hepatitis (2)		Routine lab work (CBC, Chem, UA) + AUS + Liver biopsy (2)
Allergic dermatitis (2)		Characteristic clinical presentation + rule out endoparasites + dermatologic exam (2)
Pancytopenia (1)		Routine lab work (CBC, Chem, UA) + infectious disease testing (tick borne disease panel) + Bone marrow biopsy (1)

Abbreviations: AUS, abdominal ultrasonography; BMA, bone marrow aspiration; BMB, bone marrow biopsy; CSF, cerebrospinal fluid; DLE, discoid lupus erythematosus; EMG, electromyography; IBD, inflammatory bowel disease; IMHA, immune-mediated hemolytic anemia; IMNP, immune-mediated neutropenia; IMPA, immune-mediated polyarthritis; ITP, immune-mediated thrombocytopenia; MRI, magnetic resonance imaging; PLN, protein losing nephropathy; SLE, systemic lupus erythematosus; TXR, thoracic radiographs; UPC, urine protein creatinine ratio;

*The diagnostic tests listed are not a comprehensive list of all diagnostics performed but rather those instrumental in determining the definitive diagnosis.
